# Cosmetic satisfaction and patient-reported outcome measures following cranioplasty after craniectomy – A prospective cohort study

**DOI:** 10.1016/j.bas.2023.101767

**Published:** 2023-06-18

**Authors:** Vita M. Klieverik, Pierre A. Robe, Marvick S.M. Muradin, Peter A. Woerdeman

**Affiliations:** a Department of Neurology and Neurosurgery, University Medical Center Utrecht, Utrecht, the Netherlands; b Department of Oral and Maxillofacial Surgery, University Medical Center Utrecht, Utrecht, the Netherlands

**Keywords:** Cosmesis, Patient-reported outcomes, Cranioplasty, Craniectomy

## Abstract

**Introduction:**

Evaluating patient-reported outcomes (PROMs) helps optimize preoperative counseling and psychosocial care for patients who underwent cranioplasty.

**Research question:**

This study aimed to evaluate cosmetic satisfaction, level of self-esteem, and fear of negative evaluation (FNE) of patients who underwent cranioplasty.

**Material and methods:**

Patients who underwent cranioplasty from 1 January 2014 to 31 December 2020 ​at University Medical Center Utrecht and a control group consisting of our center’ employees were invited to fill out the Craniofacial Surgery Outcomes Questionnaire (CSO-Q), consisting of an assessment of cosmetic satisfaction, the Rosenberg Self-Esteem Scale (RSES), and the FNE scale. To test for differences in results, chi-square tests and T-tests were performed. Logistic regression was used to study the effect of cranioplasty-related variables on cosmetic satisfaction.

**Results:**

Cosmetic satisfaction was seen in 44/80 patients (55.0%) and 52/70 controls (74.3%) (p ​= ​0.247). Thirteen patients (16.3%) and 8 controls (11.4%) had high self-esteem (p ​= ​0.362), 51 patients (63.8%) and 59 controls (84.3%) had normal self-esteem (p ​= ​0.114), and 7 patients (8.8%) and 3 controls (4.3%) had low self-esteem (p ​= ​0.337). Forty-nine patients (61.3%) and 39 controls (55.7%) had low FNE (p ​= ​0.012), 8 patients (10.0%) and 18 controls (25.7%) had average FNE (p ​= ​0.095), and 6 patients (7.5%) and 13 controls (18.6%) had high FNE (p ​= ​0.215). Cosmetic satisfaction was associated with glass fiber-reinforced composite implants (OR 8.20, p-value ​= ​0.04).

**Discussion and conclusion:**

This study prospectively evaluated PROMs following cranioplasty, for which we found favorable results.

## Highlights

• The majority of patients who underwent cranioplasty following craniectomy were satisfied or very satisfied with the aesthetic appearance of their face and skull.
• The majority of these patients also reported to have normal to high self-esteem and a low fear of negative evaluation.
• Of all studied aspects of cosmetic satisfaction, patients were the least satisfied with their head symmetry, likely due to the unilateral surgical site that may result in temporal hollowing and subsequent residual asymmetry of the head.
• Patients' cosmetic satisfaction, self-esteem and fear of negative evaluation were similar to our controls', which illustrates a limited negative impact of craniectomy and cranioplasty on these outcomes.

## 1 Introduction

Craniectomy is a potentially life-saving neurosurgical procedure performed to decrease medically refractory elevated intracranial pressure (ICP) resulting from traumatic brain injury (TBI), vascular disease, or various other conditions (Brown and Wijdicks, 2017). With advances in medical and surgical care, more patients survive their initial insult and require subsequent cranioplasty to protect the dura and brain from physical insult and to restore cosmesis. Cranioplasty also contributes to neurological recovery by reversing abnormalities in cerebral blood flow, cerebrospinal fluid (CSF) hydrodynamics, and cerebral metabolic activity (Fodstad et al., 1984; Winkler et al., 2000). Although reconstruction of normal cranial vault geometry to restore cosmesis is an important indication for cranioplasty, evaluation of patients’ postoperative cosmetic satisfaction is rarely reported in the literature (Satapathy et al., 2019). This is likely due to the general opinion that cosmetic satisfaction is less important than functional neurological recovery, especially in patients with poor neurological outcomes following craniectomy (Satapathy et al., 2019). However, a retained cosmetic deformity may negatively influence patients’ level of self-esteem, sense of belonging, social behavior, and overall health-related quality of life (Salokorpi et al., 2019). Therefore, it is important to evaluate different patient-reported outcome measures (PROMs) following cranioplasty, including patients’ cosmetic satisfaction, self-esteem, and fear of negative evaluation (FNE). These results help provide insight into how cranioplasty following craniectomy affects patients’ lives, which may help optimize preoperative counseling and psychosocial care for these patients and their families. Therefore, the objective of the present study is 1) to evaluate long-term cosmetic satisfaction and other PROMs of patients who underwent cranioplasty following craniectomy and 2) to provide an inclusive and uniform tool for assessing these outcomes.

## 2 Methods

### 2.1 Study design and study population

We identified all consecutive patients who underwent cranioplasty following craniectomy from 1 January 2014 to 31 December 2020 ​at the University Medical Center Utrecht, the Netherlands. Patients were eligible for inclusion in the cohort if a minimum of one-year follow-up data was available. Patients were excluded from the cohort if they underwent cranioplasty for the treatment of craniostenosis or craniosynostosis. This study was reviewed and approved by our institution’s Medical Research Ethics Committee (MREC) under number 22/519 and all included patients provided informed consent.

### 2.2 Assessment of cosmetic satisfaction and other PROMs

We prospectively collected data regarding cosmetic satisfaction and other PROMs using the Craniofacial Surgery Outcomes Questionnaire (CSO-Q). All patients in the cohort over the age of 18 years were invited to fill out the CSO-Q after a minimum of one-year follow-up after cranioplasty. The CSO-Q was distributed in April 2022 using the electronic data capture (EDC) tool Castor and closed in October 2022 (Castor, 2019). An e-mail was sent to all eligible patients in the cohort, detailing the objective of the study, background information, an informed consent form, and a link to the CSO-Q. All responses were completed anonymously.

The introductory part of the CSO-Q contains questions regarding demographic information, including patients’ general health and social situation. Part A of the CSO-Q includes an assessment of patients’ satisfaction with the aesthetic appearance of the face and skull. This assessment contains 6 questions which can be answered on a 5-point Likert scale (very satisfied, 4 points; satisfied, 3 points; neutral, 2 points; not satisfied, 1 point; and not satisfied at all, 0 points). Part B of the CSO-Q consists of the Rosenberg Self-Esteem Scale (RSES), the most widely used and validated scale to measure individuals’ level of self-esteem (Rosenberg, 1965). The RSES contains 10 statements for which patients need to decide whether they agree or disagree using a 4-point Likert scale (strongly agree, 3 points; agree, 2 points; disagree, 1 point; strongly disagree, 0 points). Statements 3, 5, 8, 9, and 10 are reverse scored. Part C of the CSO-Q includes an assessment of patients’ feelings of noticeability of their facial and skull appearance to others. This assessment contains 3 questions which can be answered on a 3-point Likert scale (often, 2 points; sometimes, 1 point; never, 0 points). Part D of the CSO-Q consists of the FNE scale, a standardized and validated tool to measure anxiety associated with perceived negative evaluation (Watson and Friend, 1969). The FNE scale contains 30 true-false statements of which 17 are straightforwardly-worded (directly scored) and 13 are reverse-worded (reverse scored). The full CSO-Q is provided in the Supplementary Material.

To compare the patients’ results of the CSO-Q to those of persons without a medical history of craniofacial or neurological surgery, we invited employees of our center to participate in a control group and also fill out the CSO-Q. We distributed the CSO-Q on 1 December 2022 to all of our employees using our center’s intranet and closed it on 31 December 2022. An announcement was placed on the homepage of our center’s intranet, detailing the objective of the study, background information, and a link to the CSO-Q. To adjust the CSO-Q to fit a control group without a medical history of craniofacial or neurological surgery, we only included part A (excluding questions 5 and 6), B, and D. All our employees’ responses were completed anonymously.

### 2.3 Statistical analysis

A descriptive analysis of the results of the CSO-Q was performed. Normally distributed continuous data were presented as means ​± ​standard deviations (SD). Skewed distributed continuous data were expressed as medians with corresponding interquartile ranges (IQR). Categorical data were shown as numbers with corresponding percentages. Likert-scale data of the patients were visualized using stacked bar charts.

Results of part A of the CSO-Q were dichotomized into cosmetic satisfaction (very satisfied or satisfied with 1) at least 4 out of 6 questions for the patients or 2) at least 3 out of 4 questions for the controls) and cosmetic dissatisfaction. Based on the results of the RSES, patients and controls were categorized into high self-esteem (score of 26–30 points), normal self-esteem (score of 15–25 points), and low self-esteem (score of <15 points). Based on the results of the FNE scale, patients and controls were grouped into low fear (score of ≤12 points), average fear (score of 13–20 points), and high fear (score of 21–30 points). Results of the RSES and FNE scale were further dichotomized into good self-esteem (high and normal self-esteem combined) versus low self-esteem, and presence (high and average fear combined) versus absence of FNE, respectively.

To test for differences in the proportions of the different results of the CSO-Q between patients and controls, chi-square tests were performed. To test for differences in the means of the RSES score and the FNE scale between patients and controls, independent samples T-tests were performed.

Potential correlations between patients’ total scores of the different parts of the CSO-Q were evaluated using the Spearman correlation. To test for differences in rates of postoperative complications and revision surgeries between patients with cosmetic satisfaction versus dissatisfaction, good versus low self-esteem, and presence versus absence of FNE, we used chi-square tests and Fisher’s exact tests. Univariable and multivariable logistic regression analysis was used to study the potential effect of different cranioplasty-related clinical variables on patients’ cosmetic satisfaction. Prior examination of the literature as well as expert opinion guided the selection of the clinical variables (Satapathy et al., 2019). These included indication for craniectomy, cranial defect size, time interval between craniectomy and cranioplasty, age at cranioplasty, cranioplasty implant material, method of implant fixation, and method of skin closure. Statistical significance was defined as p-value <0.05. All statistical analyses were performed using R statistical software, version 4.0.2. (R Foundation for Statistical Computing, Vienna, Austria).

## 3 Results

### 3.1 Baseline characteristics

A total of 182 patients underwent cranioplasty following craniectomy within the study timeframe. Of this cohort, 80 patients (44.0%) returned the CSO-Q. Of these patients, 38 (47.5%) were male and the mean age at cranioplasty was 43.9 ​± ​17.5 years (range 11.9–75.6 years). The median time interval between cranioplasty and returning the CSO-Q was 5.5 years (IQR 4.2–7.0 years, range 1.6–29.8 years). The mean age at returning the CSO-Q was 50.3 ​± ​16.9 years (range 18.8–77.9 years).

A total of 70 out of 12000 employees of our center participated in the control group and also filled out the CSO-Q. Of these controls, 16 (22.9%) were male and the mean age was 36.6 ​± ​12.7 years (range 21.0–63.0 years). The baseline characteristics of patients and controls are shown in Table 1.

**Table 1:** Baseline characteristics of 80 patients and 70 controls who returned the CSO-Q (all data given as number of patients (%) unless otherwise indicated).

Characteristic	Patients	Controls
Males	38 (47.5)	16 (22.9)
Mean age at cranioplasty ​± ​SD in years	43.9 ​± ​17.5	*na*
Age distribution at cranioplasty in years		
< ​18	4 (5.0)	*na*
18–29	19 (23.8)	*na*
30–39	11 (13.8)	*na*
40–49	7 (8.8)	*na*
50–59	22 (27.5)	*na*
60–69	11 (13.8)	*na*
≥ ​70	4 (5.0)	*na*
Mean cranial defect size ​± ​SD in cm^2^	81.3 ​± ​34.4	*na*
Cranial defect size categories in cm^2^		
< ​75	25 (31.3)	*na*
≥ ​75	49 (61.3)	*na*
Cranial reconstruction material		
Autologous bone flap	52 (65.0)	*na*
Glass-fiber reinforced composite	14 (17.5)	*na*
MMA	9 (11.3)	*na*
PMMA	3 (3.8)	*na*
PEEK	2 (2.5)	*na*
Skin closure following cranioplasty		
Sutures	56 (70.0)	*na*
Staples	20 (25.0)	*na*
Median time-interval between cranioplasty and CSO-Q in years (IQR)	5.5 (4.2–7.0)	*na*
Mean age at CSO-Q ​± ​SD in years	50.3 ​± ​16.9	36.6 ​± ​12.7
Age distribution at CSO-Q in years		
18–29	13 (16.3)	30 (42.9)
30–39	14 (17.5)	18 (25.7)
40–49	10 (12.5)	6 (8.6)
50–59	14 (17.5)	14 (20.0)
60–69	19 (23.8)	2 (2.9)
≥ ​70	10 (12.5)	
Rating of general health		
Good	48 (60.0)	*na*
Fair	28 (35.0)	*na*
Bad	4 (5.0)	*na*
Educational attainment∗		
High	35 (43.8)	59 (84.3)
Intermediate	24 (30.0)	11 (15.7)
Low	16 (20.0)	0 (0.0)
Employment status		
Employed	25 (31.3)	70 (100.0)
Unemployed	52 (65.0)	0 (0.0)
Social situation		
Single	24 (30.0)	*na*
Relationship	52 (65.0)	*na*

CSO-Q = Craniofacial Surgery Outcomes Questionnaire, *na* ​= ​not applicable, SD ​= ​standard deviation, MMA ​= ​methyl methacrylate, PMMA ​= ​polymethyl methacrylate, PEEK ​= ​polyetheretherketone, IQR ​= ​interquartile range. ∗ High educational attainment ​= ​university or college degree; intermediate educational attainment ​= ​high school or vocational education degree; low educational attainment ​= ​pre-vocational education degree or no education. Missing values were present for patients for age at cranioplasty (2.5%), cranial defect size (7.5%), skin closure following cranioplasty (5.0%), time interval between cranioplasty and CSO-Q (2.5%), educational attainment (6.3%), employment status (3.8%), and social situation (5.0%).

### 3.2 Outcomes of the Craniofacial Surgery Outcomes Questionnaire

Of all patients who returned the CSO-Q, a total of 86.3% completed all questions of part A, 85.0% finished part B (the RSES), 90.0% completed part C, and 67.5% finalized part D (the FNE scale). All controls completed all parts of the CSO-Q. The differences in the results of the CSO-Q between patients and controls are summarized in Table 2.

**Table 2:** Differences in the results of the CSO-Q between patients and controls (all data given as number of patients (%) unless otherwise indicated).

Results of the CSO-Q	Patients	Controls	*p-value*
Cosmetic satisfaction	44 (55.0)	52 (74.3)	0.247
RSES			
High self-esteem	13 (16.3)	8 (11.4)	0.362
Normal self-esteem	51 (63.8)	59 (84.3)	0.114
Low self-esteem	7 (8.8)	3 (4.3)	0.337
Mean ​± ​SD	20.3 ​± ​5.1	20.7 ​± ​4.1	0.569
FNE scale			
Low FNE	49 (61.3)	39 (55.7)	0.012
Average FNE	8 (10.0)	18 (25.7)	0.095
High FNE	6 (7.5)	13 (18.6)	0.215
Mean ​± ​SD	8.2 ​± ​7.4	12.8 ​± ​6.7	<0.001

CSO-Q = Craniofacial Surgery Outcomes Questionnaire, RSES = Rosenberg Self-Esteem Scale, FNE ​= ​fear of negative evaluation, SD ​= ​standard deviation. Missing values were present for patients for cosmetic satisfaction (13.8%), the RSES (11.3%), and the FNE scale (21.3%).

Patients’ results of part A of the CSO-Q are presented in Fig. 1a, Fig. 1ba and Fig. 1b. Overall, cosmetic satisfaction was seen in 44 patients (55.0%) and 52 controls (74.3%) and cosmetic dissatisfaction in 25 patients (31.3%) and 18 controls (25.7%) (p ​= ​0.247, missing values for patients ​= ​13.8%). Fig. 2a, Fig. 2ba and Fig. 2b shows the patients’ results of the RSES. Thirteen patients (16.3%) and 8 controls (11.4%) were categorized as having high self-esteem (p ​= ​0.362), 51 patients (63.8%) and 59 controls (84.3%) were classified as having normal self-esteem (p ​= ​0.114), and 7 patients (8.8%) and 3 controls (4.3%) had low self-esteem (p ​= ​0.337, missing values for patients ​= ​11.3%). The mean RSES score was 20.3 ​± ​5.1 for patients and 20.7 ​± ​4.1 for controls (p ​= ​0.569). Patients’ answers to part C of the CSO-Q are shown in Fig. 3. Based on the results of the FNE scale, 49 patients (61.3%) and 39 controls (55.7%) were classified as having a low FNE (p ​= ​0.012), 8 patients (10.0%) and 18 controls (25.7%) showed to have an average FNE (p ​= ​0.095), and 6 patients (7.5%) and 13 controls (18.6%) were categorized as having a high FNE (p ​= ​0.215, missing values for patients ​= ​21.3%). The mean score of the FNE scale was 8.2 ​± ​7.4 for patients and 12.8 ​± ​6.7 for controls (p ​< ​0.001).

**Fig. 1a:**
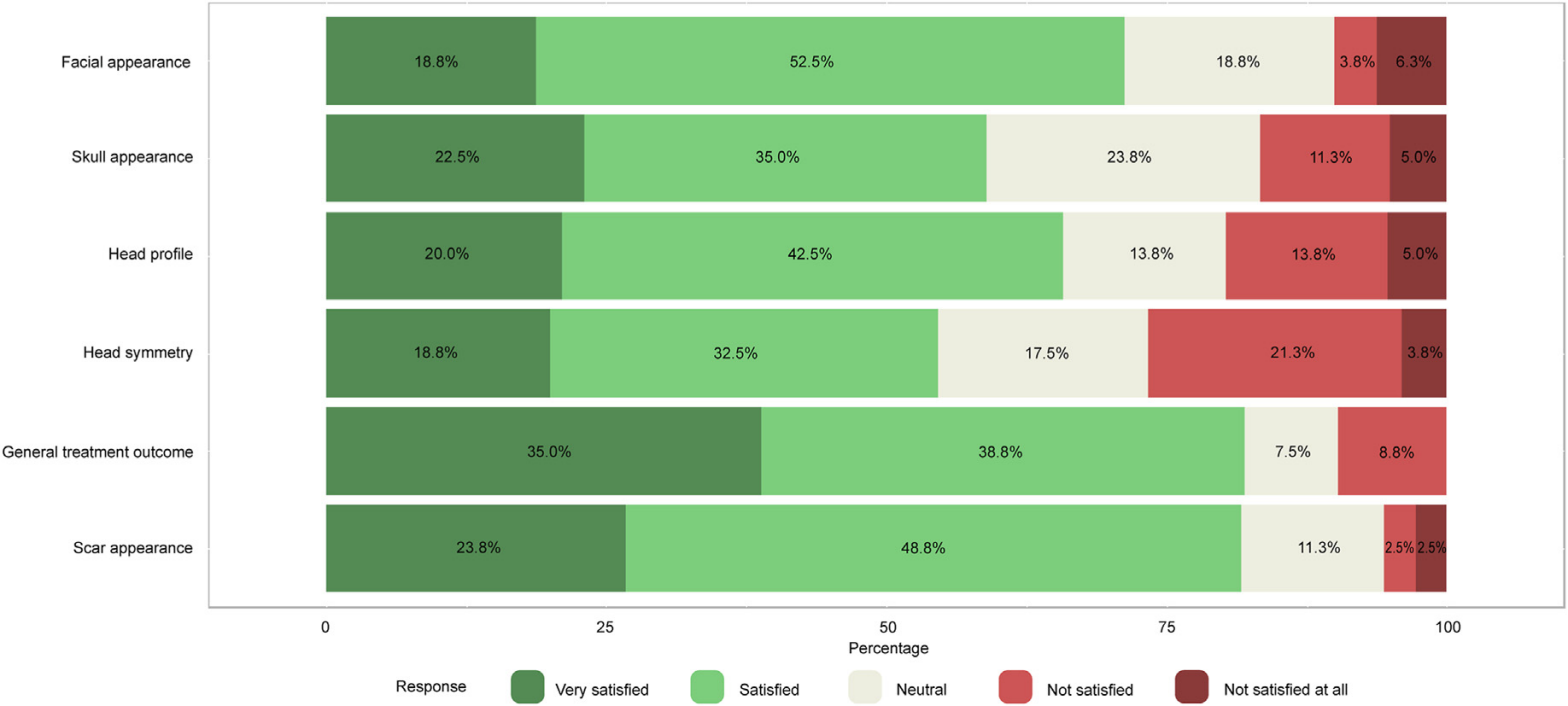
Likert scale of satisfaction with the aesthetic appearance of the face and skull of 80 patients who returned the Craniofacial Surgery Outcomes Questionnaire (CSO-Q). Missing values were present for skull appearance (2.5%), head profile (5.0%), head symmetry (6.3%), general treatment outcome (10.0%), and scar appearance (11.3%).

**Fig. 1b:**
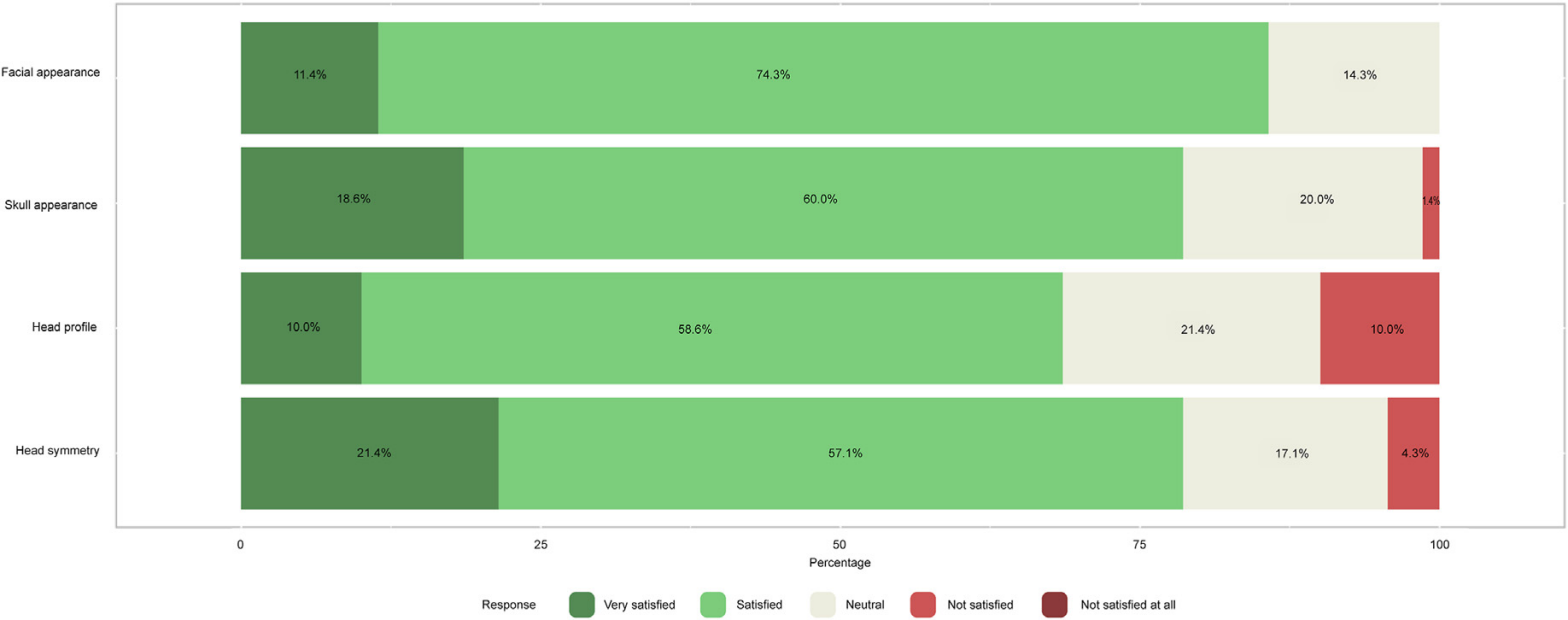
Likert scale of satisfaction with the aesthetic appearance of the face and skull of 70 employees who returned the Craniofacial Surgery Outcomes Questionnaire (CSO-Q).

**Fig. 2a:**
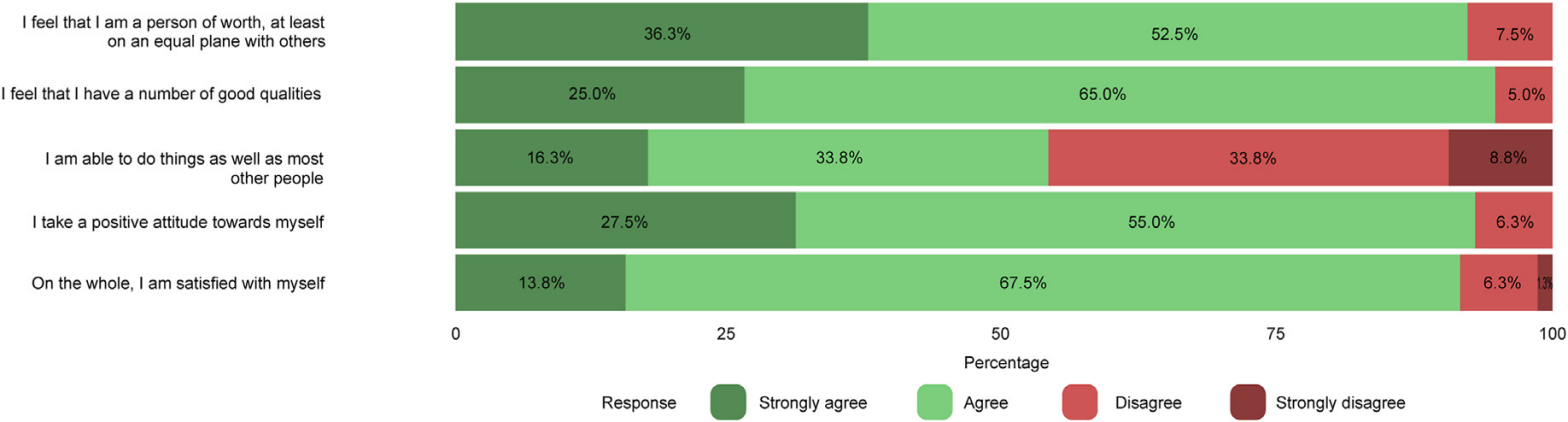
Likert scale of the straightforwardly-worded statements of the Rosenberg Self-Esteem Scale (RSES) of 80 patients who returned the Craniofacial Surgery Outcomes Questionnaire (CSO-Q). Missing values were present for statement 1 (3.8%), statement 2 (5.0%), statement 3 (7.5%), statement 4 (11.3%), and statement 5 (11.3%).

**Fig. 2b:**
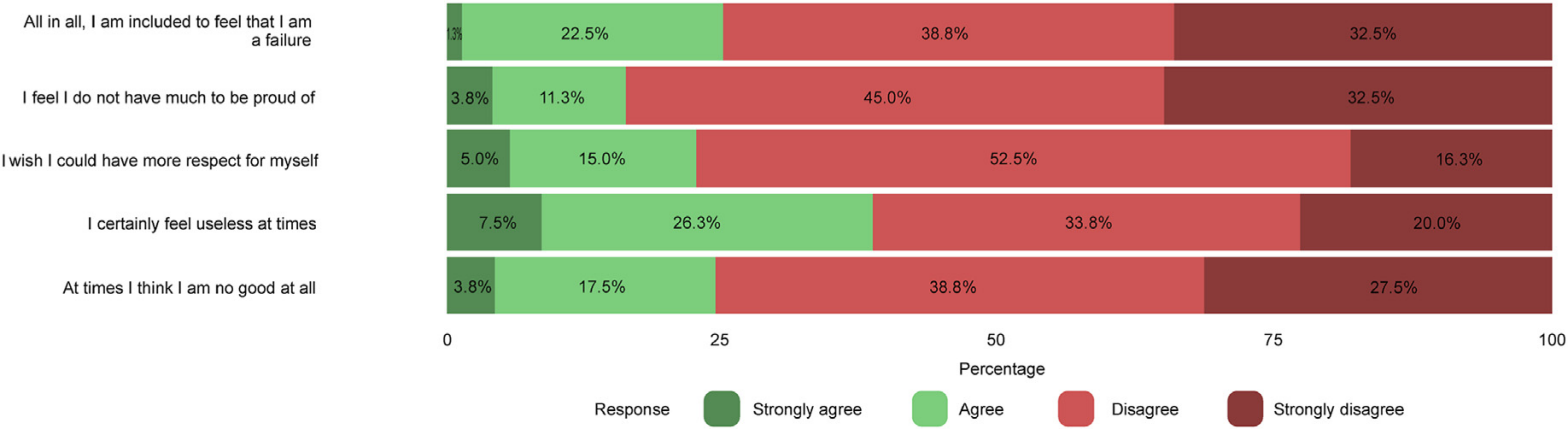
Likert scale of the reverse-worded statements of the Rosenberg Self-Esteem Scale (RSES) of 80 patients who returned the Craniofacial Surgery Outcomes Questionnaire (CSO-Q). Missing values were present for statement 3 (5.0%), statement 5 (7.5%), statement 8 (11.3%), statement 9 (12.5%), and statement 10 (12.5%).

**Fig. 3:**
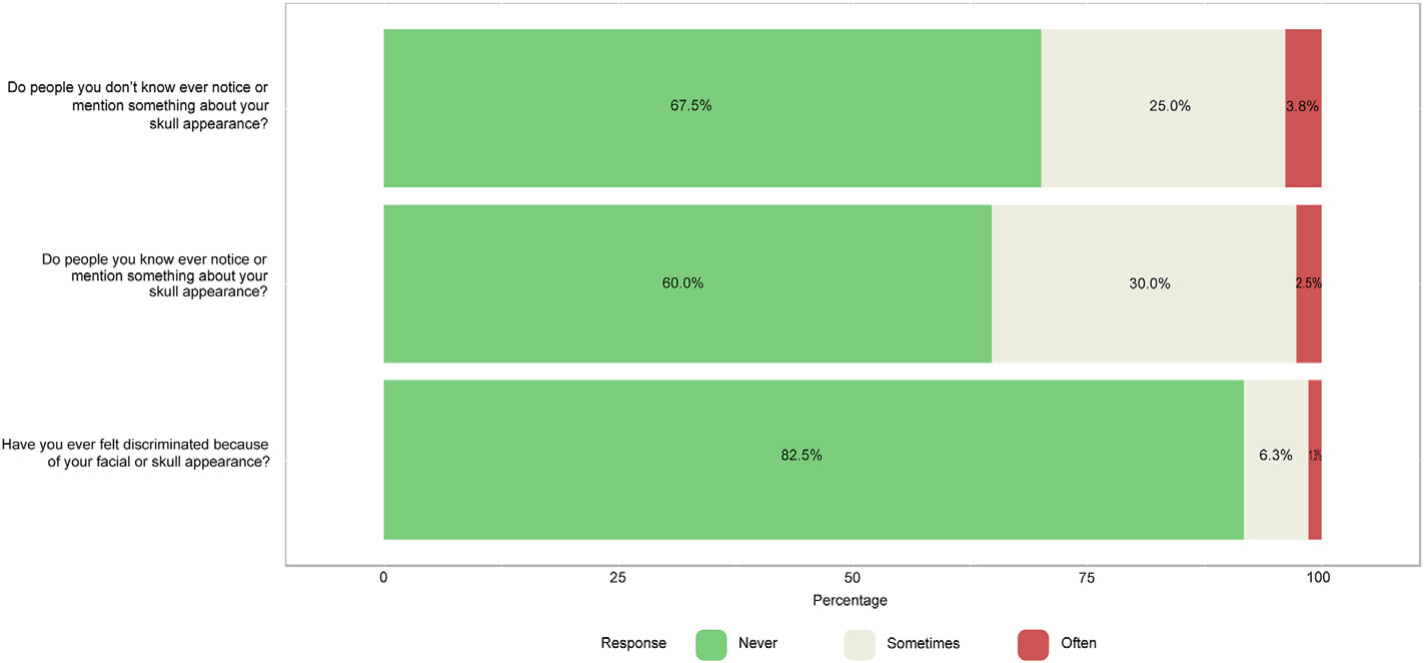
Likert scale of patients’ feelings of noticeability of facial and skull appearance to others of 80 patients who returned the Craniofacial Surgery Outcomes Questionnaire (CSO-Q). Missing values were present for question 1 (3.8%), question 2 (7.5%), and question 3 (10.0%).

For the patients who were dissatisfied with their head symmetry (i.e., the patients who scored ‘Neutral’, ‘Not satisfied’ or ‘Not satisfied at all’ on this question of part A of the CSO-Q), we examined the available postoperative imaging and studied whether the head circumference was measurably asymmetric in these patients by comparing the circumference of both sides of the skull contour. A total of 34 patients (42.5%) were dissatisfied with their head symmetry and 17 of them (50.0%) underwent postoperative imaging (computed tomography [CT] scans in 15 patients and magnetic resonance imaging [MRI] in 2 patients). Of these patients, the head circumference was measurably asymmetric in 9 patients (52.9%) and the difference in circumference between the sides of the skull contour ranged from 4.0 to 24.0 ​mm.

### 3.3 Correlations between the different PROMs

We found a statistically significant positive correlation between patients’ total score of part A and of the RSES (Spearman’s rank correlation coefficient ​= ​0.24, p-value ​= ​0.04). A statistically significant negative correlation was found between the total scores of part A and part C (Spearman’s rank correlation coefficient ​= ​−0.41, p-value <0.001), and between the total scores of the RSES and the FNE scale (Spearman’s rank correlation coefficient ​= ​−0.44, p-value <0.001).

### 3.4 Differences in rates of postoperative complications and revision surgeries

We did not find statistically significant differences in rates of postoperative complications and revision surgeries between patients with cosmetic satisfaction versus dissatisfaction, good versus low self-esteem, and presence versus absence of FNE, respectively (Table 3a, Table 3ba and Table 3b).

**Table 3a:** Differences in rates of postoperative complications and revision surgeries between patients with cosmetic satisfaction versus dissatisfaction (all data given as number of patients (%) unless otherwise indicated).

Event	Cosmetic satisfaction (n ​= ​44)	Cosmetic dissatisfaction (n ​= ​25)	p-value∗
Total patients with complication	18 (40.9)†	9 (36.0)†	0.688
Bone flap resorption	12 (27.3)	7 (28.0)	0.948
Surgical site infection	6 (13.6)	3 (12.0)	0.846
Mechanical complications	1 (2.3)	0 (0.0)	1.000
First revision surgery	18 (40.9)	7 (28.0)	0.284

∗ Chi-square test or Fisher’s exact test (for cell count less than five) for difference in proportions.

† In both groups, 1 patient experienced both bone flap resorption and surgical site infection.

**Table 3b:** Differences in rates of postoperative complications and revision surgeries between patients with good self-esteem versus low self-esteem (all data given as number of patients (%) unless otherwise indicated).

Event	Good self-esteem (n ​= ​64)	Low self-esteem (n ​= ​7)	p-value∗
Total patients with complication	27 (42.2)†	2 (28.6)	0.487
Bone flap resorption	20 (31.3)	1 (14.3)	0.350
Surgical site infection	9 (14.1)	1 (14.3)	0.987
Mechanical complications	1 (1.6)	0 (0.0)	1.000
First revision surgery	25 (39.1)	2 (28.6)	0.587

∗ Chi-square test or Fisher’s exact test (for cell count less than five) for difference in proportions.

† 2 patients experienced both bone flap resorption and surgical site infection and 1 patient experienced both bone flap resorption and mechanical complications.

### 3.5 Clinical variables associated with cosmetic satisfaction

Univariable analysis identified a trend associating cosmetic satisfaction in patients with the use of glass fiber-reinforced composite as the cranioplasty implant material Table 3c (OR 4.02, p-value ​= ​0.09). This association reached statistical significance on multivariable analysis (OR 8.20, p-value ​= ​0.04) when corrected for indication for craniectomy, cranial defect size, time interval between craniectomy and cranioplasty, age at cranioplasty, cranioplasty implant material, method of implant fixation, and method of skin closure (Table 4). We did not find statistically significant associations between cosmetic satisfaction in patients and any of the other cranioplasty-related clinical variables.

**Table 3c:** Differences in rates of postoperative complications and revision surgeries between patients with presence versus absence of fear of negative evaluation (all data given as number of patients (%) unless otherwise indicated).

Event	Presence of fear of negative evaluation (n ​= ​14)	Absence of fear of negative evaluation (n ​= ​49)	p-value∗
Total patients with complication	3 (21.4)	19 (38.8)	0.230
Bone flap resorption	2 (14.3)	14 (28.6)	0.279
Surgical site infection	2 (14.3)	6 (12.2)	0.840
Mechanical complications	0 (0.0)	1 (2.0)	1.000
First revision surgery	2 (14.3)	18 (36.7)	0.112

^†^ 2 patients experienced both bone flap resorption and surgical site infection and 1 patient experienced both bone flap resorption and mechanical complications.

∗ Chi-square test or Fisher’s exact test (for cell count less than five) for difference in proportions.

**Table 4:** Univariable and multivariable logistic regression analysis of clinical variables affecting cosmetic satisfaction.

	Univariable		Multivariable	
Variable	OR	p-value	OR	p-value
Indication for craniectomy				
Trauma	Reference			
Vascular disease	0.65	0.48	0.88	0.86
Infection	0.58	0.38	0.44	0.38
Cranial defect size (per cm^2^)	1.00	0.75	1.01	0.48
Time interval craniectomy and cranioplasty (per day)	1.00	0.80	1.00	0.86
Age at cranioplasty (per year)	1.00	0.91	0.99	0.56
Cranioplasty implant material				
Autologous bone graft	Reference			
MMA	1.46	0.68	6.91	0.21
PMMA	1.46	0.76	1.48	0.81
PEEK	0.73	0.83	0.85	0.92
Glass fiber-reinforced composite	4.02	0.09	8.20	0.04
Method of implant fixation				
Screws and plates	Reference			
Sutures	0.56	0.68	1.48	0.82
Method of skin closure				
Sutures	Reference			
Staples	2.99	0.12	3.29	0.13

OR ​= ​odds ratio, cm ​= ​centimeter, MMA ​= ​methyl methacrylate, PMMA ​= ​polymethyl methacrylate, PEEK ​= ​polyetheretherketone.

## 4 Discussion

The present study prospectively and systematically evaluated an extensive assortment of PROMs following cranioplasty, including patients’ perceived cosmetic satisfaction, level of self-esteem, and extent of FNE. Overall, we found favorable results of the evaluated PROMs. The majority (55.0%) of patients were satisfied or very satisfied with the aesthetic appearance of their face and skull and reported to have normal to high self-esteem and low FNE. Compared to the other aspects of cosmetic satisfaction, patients were the least satisfied with their head symmetry, likely due to the unilateral surgical site that may result in temporal hollowing and subsequent residual asymmetry of the head. We found that in the majority of the patients who were dissatisfied with their head symmetry, the head circumference was also measurably asymmetric on postoperative imaging. These results emphasize the need to restore harmonious cranial symmetry in these patients as head symmetry seems an important factor for cosmetic satisfaction. Few studies have focused on cosmetic satisfaction as perceived by patients themselves and those that did lacked a detailed assessment of the different aspects of cosmetic satisfaction (Baldia et al., 2022; Nguyen et al., 2021; Henker et al., 2018; Lannon et al., 2022; Morales-Gómez et al., 2018; Giese et al., 2021; Moles et al., 2018; Fischer et al., 2012; Asaad et al., 2021). Nevertheless, these studies reported overall similar results, with the majority of patients reporting satisfaction with their aesthetic appearance.

Furthermore, we found that the results of the evaluated PROMs were similar in our patients and our controls, which illustrates a limited negative impact of craniectomy and cranioplasty on patients’ cosmetic satisfaction, self-esteem, and FNE. The results were similar even despite the differences in age, educational attainment and employment status between our patients and controls. In fact, we found that the extent of FNE was significantly lower in our patients than in our controls. This result suggests that the extent of FNE is not affected by craniectomy and cranioplasty. Moreover, we found that the mean scores of the RSES and FNE scale of our patients and our controls are comparable with the mean scores of studies conducted in the general population of the United States and France (Sinclair et al., 2010; Musa et al., 2004). Self-esteem is defined as a persons’ overall sense of worth as an individual, while FNE is characterized as the experience of distress associated with negative evaluation from others (Rosenberg, 1965; Watson and Friend, 1969). In general, different factors may affect individual persons’ level of self-esteem and extent of FNE, such as their age, personality traits, psychological resilience, socioeconomic status, social relationships, and cultural context (Von Soest et al., 2018; Zeigler-Hill et al., 2015; Harris and Orth, 2020; Fulmer et al., 2010; Zimmermann et al., 2020; Javida et al., 2021). In cranioplasty patients, additional factors may have an effect on these PROMs, including indication for craniectomy, neurological outcomes following craniectomy, and patients’ degree of coping with physical and functional disability and rehabilitation (Curvis et al., 2018). For example, patients who have survived TBI may have difficulty accepting the post-injury representations of the self (Curvis et al., 2018; Ponsford et al., 2014). Still, our results suggest that patients’ level of self-esteem and extent of FNE remain favorable after cranioplasty following craniectomy. Although these PROMs might not necessarily be affected by surgical advancements of cranioplasty, they remain important to assess to allow for improvements in adequate psychosocial care for these patients.

We identified significant correlations between the different PROMs. We found that if patients were more satisfied with the aesthetic appearance of the face and skull, they were more likely to have a higher level of self-esteem and less likely to feel that their facial and skull appearance is noticeable to others. The significant correlation between the total scores of the RSES and FNE scale suggest that if patients reported a higher level of self-esteem, they were more likely to have a lower FNE.

Moreover, we found that cosmetic satisfaction was associated with the use of glass fiber-reinforced composite as the cranioplasty implant material. These cranial implants are relatively new and are developed to resemble the structure and properties of autologous bone grafts (Piitulainen et al., 2019). Different studies have reported on the safety and biocompatibility of these cranial implants in both adult and pediatric patient populations, concluding they may be considered as a safe and feasible cranioplasty implant material (Piitulainen et al., 2015a, 2015b, 2019; Aitasalo et al., 2014). Our results suggest that the use of glass fiber-reinforced composite also seem to improve patients’ cosmetic satisfaction compared to using autologous bone grafts or other cranial implants.

### 4.1 Implications

In general, different PROMs are increasingly used in neurosurgical practice (Ghimire et al., 2018). As clinical care transitions from disease-centered to patient-centered, evaluation of PROMs has become a priority in clinical research. Evidence shows that the systematic use of data obtained from PROMs results in enhanced communication and decision making between physicians and patients and improves patients’ satisfaction with the provided clinical care (Nelson et al., 1136; Chen et al., 2013; Marshall et al., 2006; Santana and Feeny, 2014; Valderas et al., 2008; Wasson et al., 1999). Furthermore, direct data collection from patients using PROMs limits distortion by observer bias and helps increase the public accountability of health care services (Ghimire et al., 2018).

The results of the present study provide insight into how cranioplasty following craniectomy affects patients’ lives. These data are useful for patients and their physicians to incorporate into preoperative counseling and postoperative psychosocial care, which may help anticipate patients’ concerns and improve their confidence in the provided clinical care. A uniform approach to assess PROMs following cranioplasty is currently lacking (Satapathy et al., 2019). The CSO-Q provides for an inclusive and systematic evaluation of an extensive assortment of PROMs following cranioplasty. We recommend to use the CSO-Q prior to the outpatient clinic follow-up visits to form a brief but comprehensive impression of how cranioplasty following craniectomy has affected patients’ lives. This may help offer psychosocial support resources to those patients most in need.

### 4.2 Strengths and limitations

An important strength of our study is that is the first prospective cohort study to systematically evaluate a variety of PROMs following cranioplasty. Instead of solely using physicians’ judgement of facial and skull appearance to measure cosmetic satisfaction, we studied this outcome measure as perceived by patients themselves. Moreover, we assessed how patients’ aesthetic appearance following cranioplasty might have influenced their self-esteem and FNE. For this we used the RSES and FNE scale, two reliable and valid quantitative tools that are often used in social psychology to study individual persons’ level of self-esteem and extent of FNE, respectively (Rosenberg, 1965; Watson and Friend, 1969). The response rate of the CSO-Q in the patients was fairly high (44.0%), which implies that this tool allows for use in clinical practice. Furthermore, we compared the patients’ results to those of a control group, to evaluate how the patients’ results would relate to those of persons without a medical history of craniofacial or neurosurgical surgery. Our study also has some limitations. First, questionnaire-based research inherently comes with the risk of selection bias based on non-response bias (Grimes and Schulz, 2002). It might be that patients and controls who scored higher on cosmetic satisfaction, level of self-esteem, or extent of FNE were more likely to fill out and return the CSO-Q. Second, we found that the controls who returned the CSO-Q were mostly young women with a high educational attainment, which may limit the representativeness of our control group. Nevertheless, our results still allow for drawing the useful conclusion that craniectomy and cranioplasty has a limited negative impact on the evaluated PROMs. Third, we found that as patients progressed through the CSO-Q, they stopped answering the questions, suggesting that the CSO-Q in its current form is too long for patients to complete. The resulting missing data may have introduced selection bias and caused reduced statistical power (Altman and Bland, 2007). To overcome this issue, replacing the full FNE scale with the brief FNE scale (BFNE) may be considered (Collins et al., 2005). Lastly, we may have had insufficient statistical power to detect statistically significant differences or associations in our results. To detect more potential associations between the different PROMs and rates of postoperative complications, revision surgeries, and other cranioplasty-related clinical variables, future studies with larger sample sizes are warranted.

## 5 Conclusion

The present study found favorable results of an extensive assortment of PROMs following cranioplasty, including patients’ perceived cosmetic satisfaction, level of self-esteem and extent of FNE. The presented CSO-Q provides a brief but comprehensive impression of the effect of cranioplasty following craniectomy on patients’ lives.

## Funding

This research did not receive any specific grant from funding agencies in the public, commercial, or not-for-profit sectors.

## Declaration of competing interest

The authors have no competing interests to declare.
