# Supplementary material for: Cosmetic satisfaction and patient-reported outcome measures following cranioplasty after craniectomy – A prospective cohort study
Source: Brain Spine. 2023 Jun 18;3:101767. doi: 10.1016/j.bas.2023.101767 (PMC10293317; doi:10.1016/j.bas.2023.101767)
Supplement: Multimedia component 1 [file mmc1.zip › CRANIO_spl_1.PDF]

Craniofacial Surgery Outcomes Questionnaire (CSO-Q)

Introduction

Sex:

- ◯ Male
- ◯ Female
- ◯ Prefer not to say
- ◯ Prefer to self-describe as

Age:

General health:

- ◯ Good
- ◯ Fair
- ◯ Bad

Highest degree of education achieved:

Employment:

- ◯ Yes
- ◯ No

Type of employment:

Social situation:

- ◯ Relationship
- ◯ Single

Part A: Satisfaction with the aesthetic appearance of the face and skull

1. Are you satisfied with your facial appearance?

◯ Very satisfied

◯ Satisfied

◯ Neutral

◯ Not satisfied

◯ Not satisfied at all, and wish change could somehow be made

If anything, what could be better?

Other comments:

2. Are you satisfied with your skull appearance? In other words, how the way your head is shaped?

◯ Very satisfied

◯ Satisfied

◯ Neutral

◯ Not satisfied

◯ Not satisfied at all, and wish change could somehow be made

If anything, what could be better?

Other comments:

3. Are you happy with your head profile? In other words, how happy are you with the side view of your head?

◯ Very satisfied

◯ Satisfied

◯ Neutral

◯ Not satisfied

○ Not satisfied at all, and wish change could somehow be made

If anything, what could be better?

Other comments:

4. Are you pleased with the symmetry of your head?

○ Very satisfied

○ Satisfied

○ Neutral

○ Not satisfied

○ Not satisfied at all, and wish change could somehow be made

If anything, what could be better?

Other comments:

5. In general, how satisfied are you with the treatment outcome?

○ Very satisfied

○ Satisfied

○ Neutral

○ Not satisfied

○ Not satisfied at all, and wish change could somehow be made

If anything, what could be better?

Other comments:

6. If operated, are you satisfied with the scar healing on your skull?

○ Very satisfied

○ Satisfied

○ Neutral

○ Not satisfied

○ Not satisfied at all, and wish change could somehow be made

If anything, what could be better?

Other comments:

Part B: Rosenberg Self-Esteem Scale

STATEMENT		Strongly Agree	Agree	Disagree	Strongly Disagree
1.	I feel that I am a person of worth, at least on an equal plane with others.	○	○	○	○
2.	I feel that I have a number of good qualities.	○	○	○	○
3.	All in all, I am inclined to feel that I am a failure.	○	○	○	○
4.	I am able to do things as well as most other people.	○	○	○	○
5.	I feel I do not have much to be proud of.	○	○	○	○
6.	I take a positive attitude toward myself.	○	○	○	○
7.	On the whole, I am satisfied with myself.	○	○	○	○
8.	I wish I could have more respect for myself.	○	○	○	○
9.	I certainly feel useless at times.	○	○	○	○
10.	At times I think I am no good at all.	○	○	○	○

Part C: Feelings of noticeability of facial and skull appearance to others

1. Do people you don't know ever notice or mention something about your skull appearance?

- Never
- Sometimes
- Often

If so, what do you do or what does it do to you?

Comments:

2. Do people you know ever notice or mention something about your skull appearance?

- Never
- Sometimes
- Often

If so, what do you do or what does it do to you?

Comments:

3. Have you ever felt discriminated because of your facial or skull appearance?

- Never
- Sometimes
- Often

If so, under what kind of circumstances did that occur?

Comments:

Part D: Fear of negative evaluation

Carefully read each of the 30 statements listed below. Decide whether each statement is true or false as it pertains to you personally. If you are unsure which is the better answer, decide which one is slightly more applicable to how you are feeling at the moment and answer accordingly. Try to answer based on your first reaction to the statement. Don't spend too long on any one item.

1. I rarely worry about seeming foolish to others.

○ True

○ False

2. I worry about what people will think of me even when I know it doesn't make any difference.

○ True

○ False

3. I become tense and jittery if I know someone is sizing me up.

○ True

○ False

4. I am unconcerned even if I know people are forming an unfavorable impression of me.

○ True

○ False

5. I feel very upset when I commit some social error.

○ True

○ False

6. The opinions that important people have of me cause me little concern.

○ True

○ False

7. I am often afraid that I may look ridiculous or make a fool of myself.

◯ True

◯ False

8. I react very little when other people disapprove of me.

◯ True

◯ False

9. I am frequently afraid of other people noticing my shortcomings.

◯ True

◯ False

10. The disapproval of others would have little effect on me.

◯ True

◯ False

11. If someone is evaluating me I tend to expect the worst.

◯ True

◯ False

12. I rarely worry about what kind of impression I am making on someone.

◯ True

◯ False

13. I am afraid that others will not approve of me.

◯ True

◯ False

14. I am afraid that people will find fault with me.

- ◯ True
- ◯ False

15. Other people's opinions of me do not bother me.

- ◯ True
- ◯ False

16. I am not necessarily upset if I do not please someone.

- ◯ True
- ◯ False

17. When I am talking to someone, I worry about what they may be thinking about me.

- ◯ True
- ◯ False

18. I feel that you can't help making social errors sometimes, so why worry about it.

- ◯ True
- ◯ False

19. I am usually worried about what kind of impression I make.

- ◯ True
- ◯ False

20. I worry a lot about what my superiors think of me.

- ◯ True
- ◯ False

21. If I know someone is judging me, it has little effect on me.

- ◯ True
- ◯ False

22. I worry that others will think I am not worthwhile.

- ◯ True
- ◯ False

23. I worry very little about what others may think of me.

- ◯ True
- ◯ False

24. Sometimes I think I am too concerned with what other people think of me.

- ◯ True
- ◯ False

25. I often worry that I will say or do the wrong things.

- ◯ True
- ◯ False

26. I am often indifferent to the opinions others have of me.

- ◯ True
- ◯ False

27. I am usually confident that others will have a favorable impression of me.

- ◯ True
- ◯ False

28. I often worry that people who are important to me won't think very much of me.

◯ True

◯ False

29. I brood about the opinions my friends have about me.

◯ True

◯ False

30. I become tense and jittery if I know I am being judged by my superiors.

◯ True

◯ False

This is the end of the questionnaire. Thank you!
